# Preparing to unwind

**DOI:** 10.7554/eLife.02618

**Published:** 2014-04-01

**Authors:** Jin Chuan Zhou, Alessandro Costa

**Affiliations:** 1 **Jin Chuan Zhou** is at the London Research Institute, Cancer Research UK, Clare Hall Laboratories, London, United Kingdom; 2 **Alessandro Costa** is at the London Research Institute, Cancer Research UK, Clare Hall Laboratories, London, United Kingdomalessandro.costa@cancer.org.uk

**Keywords:** DNA replication, helicase, crystallography, biochemistry, archaea, *S. cerevisiae*, other

## Abstract

A combination of protein crystallography and biochemistry has revealed how a ring-shaped helicase might trap a single DNA strand as the double helix melts, and before it is unwound.

**Related research article** Froelich CA, Kang S, Epling LB, Bell SP, Enemark EJ. 2014. A conserved MCM single-stranded DNA binding element is essential for replication initiation. *eLife* **3**:e01993. doi: 10.7554/eLife.01993

**Image** Double-stranded DNA being unwound by helicase rings moving in opposite directions

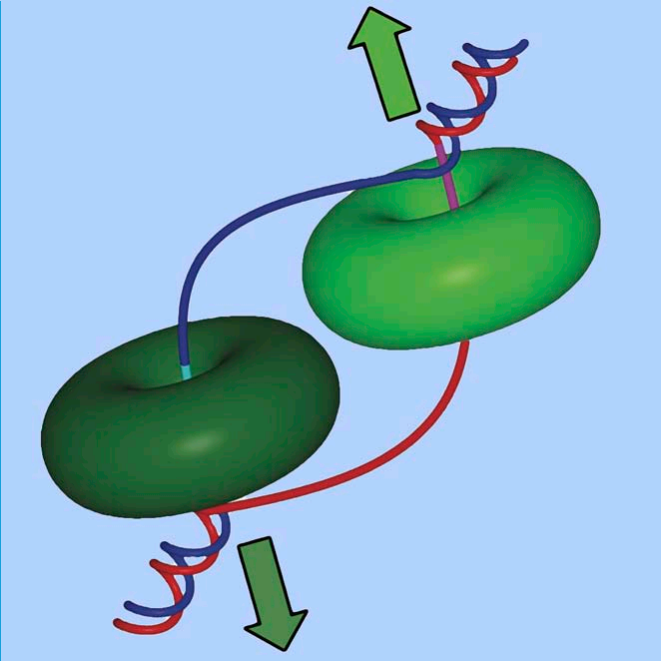

Life begets life: in order to propagate itself, a living organism must replicate its genetic material and pass this on to the next generation. To duplicate a molecule of double-stranded DNA within a cell, an ‘initiation factor’ recognises a stretch of DNA called an origin of replication and then recruits an enzyme called a helicase that goes on to unwind the double helix. This process exposes the two strands of DNA and allows them to act as templates to guide the synthesis of two new strands of DNA—which are called the leading and lagging strands. The end result is two identical molecules of double-stranded DNA.

All helicases involved in DNA replication are shaped like a ring made of six subunits and they work by selecting, encircling and sliding (or translocating) along a single strand of DNA, while keeping the other strand on the outside (Soultanas, 2012). Now, in *eLife*, researchers at St. Jude Children’s Research Hospital and the Massachusetts Institute of Technology (MIT) show how the leading strand template becomes selected as the ‘translocation strand’ by a helicase at a molecular level (Froelich et al., 2014).

Although DNA replication is vital to living things, the mechanisms behind the steps in this process are far from conserved in the different domains of life. In bacteria, for example, two copies of the helicase are loaded as single rings onto an already separated DNA duplex, and they immediately start moving in opposite directions along the lagging strands to unwind the DNA (Mott and Berger, 2007).

In eukaryotes, on the other hand, things happen more slowly. The helicase complex—called ‘Minichromosome Maintenance 2-7 complex’ or Mcm2-7 for short—also forms a ring made of six subunits (Figure 1A). However, two complexes are loaded onto the DNA as a double ring, and the rings only start moving along the leading strand templates after multiple chemical modifications have been made and various other activating factors have been recruited (Remus and Diffley, 2009; Remus et al., 2009; Fu et al., 2011).

**Figure 1.**
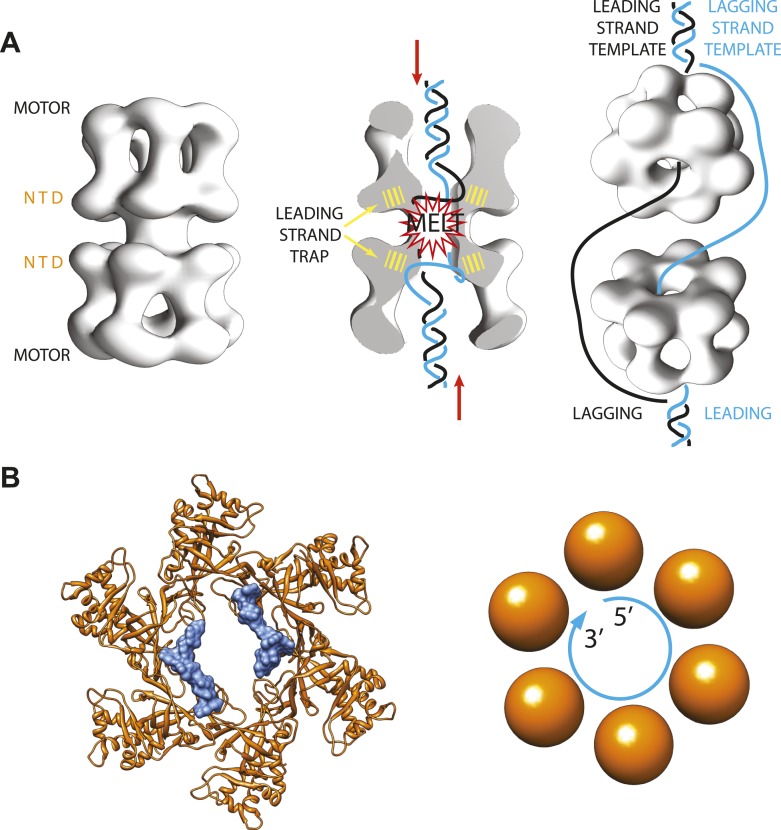
Archaeal/eukaryotic helicase interacting with DNA. (**A**) DNA opening and unwinding by the Mcm2-7 helicase: the two motor domains that move along the DNA are at opposite ends of a DNA-loaded helicase double ring, with the N-terminal DNA-binding domains (NTD) in the middle. Froelich, Kang et al. propose that the motor domains push the duplex DNA towards the middle of the helicase, hence promoting melting of the DNA at the origin of replication and trapping of the leading strand template by the NTD. The rings then separate and travel in opposite directions, with one ring sliding along each of the leading strand templates from the two replication forks. (**B**) Crystal structure of the MCM helicase (orange) bound to single-stranded DNA (light blue). The DNA circles around the MCM central channel in a clockwise direction (when travelling from the 5′-end to 3′-end and viewing the ring from its C-terminal face).

Three events must occur in order to activate the origin of replication in eukaryotes: the two Mcm2-7 rings must separate; each ring must trap the leading strand template; and each ring must eject the lagging strand template*.* However, we know very little about the details of any of these three steps in eukaryotes. And we know even less about them in archaea, although we do know that copies of proteins involved in DNA replication found in this domain of life are related to those found in eukaryotes.

Eric Enemark of St. Jude, Stephen Bell of MIT and co-workers—including Clifford Froelich and Sukhyun Kang as joint first authors—have used a combination of protein crystallography and biochemistry to shed light on the process that activates the origin of replication (Froelich et al., 2014). The Enemark lab determined the three-dimensional structure of a single-stranded fragment of DNA bound to a helicase ring from a species of archaea that lives in deep ocean vents. This helicase, called MCM, is the archaeal equivalent of the eukaryotic Mcm2-7 complex, and is easier to study because it is made of six identical protein subunits, whereas Mcm2-7 is made of six different protein subunits. Nevertheless, the two complexes are thought to be similar enough that general conclusions might be translated from one complex to the other.

As expected, the DNA binds inside the centre of the MCM ring, but it follows a different path from the DNA seen in previous helicase-DNA complex structures (Enemark and Joshua-Tor, 2006; Itsathitphaisarn et al., 2012). In fact, in the new MCM structure solved by the Enemark lab the DNA does not run straight through the ring channel but rather circles around its interior (Figure 1B). Closer inspection suggests that the N-terminal DNA-binding domain of the MCM helicase—which is the only portion of the protein present in the crystal structure—can only trap the leading (rather than lagging) strand template. This is also in agreement with previously published studies (McGeoch et al., 2005; Barry et al., 2007; Bochman and Schwacha, 2008).

Interestingly, the diameter of the MCM-bound DNA circle is much larger than that of a DNA double helix, and the points of contact between the DNA and the proteins involve sites that are usually protected in the duplex form of DNA. This suggests that the new MCM-DNA crystal structure reported by Froelich, Kang et al. might have captured an intermediate form of DNA that exists when the origin of replication melts at the onset of DNA replication (Figure 1A). This notion is also compatible with the results of in vitro experiments with related proteins from yeast, which were conducted in the Bell lab at MIT (Froelich et al. 2014).

The MCM residues that contact the DNA in the archaeal structure are fully conserved only in three of the six subunits of the eukaryotic protein complex. Remarkably, all these residues are close to one another and constitute an extended single-stranded-binding surface on the Mcm2-7 that is proposed to function in DNA melting and strand selection. A combined mutant of these three subunits can, in fact, still be loaded onto double-stranded DNA, albeit with low efficiency. However, the mutant cannot bind single stranded DNA, does not recruit some of the factors that are essential for origin activation, and it cannot support DNA replication and cell division.

Altogether, this study provides new insights into the mechanisms that activate the origin of replication in eukaryotes by identifying a role for the Mcm2-7 helicase in the processes of strand selection and, probably, origin melting. Froelich, Kang et al. propose that the two Mcm2-7 motor domains must play an important function in the early stages of origin activation, pumping duplex DNA towards the centre of the complex, and hence promoting duplex melting and the subsequent capture of the leading strand by the helicase. Determining how the Mcm2-7 motor domain engages with DNA is an important challenge for future studies.
